# Supplementary material for: Mutations in PCYT2 disrupt etherlipid biosynthesis and cause a complex hereditary spastic paraplegia
Source: Brain. 2019 Oct 22;142(11):3382–97. doi: 10.1093/brain/awz291 (PMC6821184; doi:10.1093/brain/awz291)
Supplement: awz291_Supplementary_Data [file awz291_supplementary_data.zip › awz291-Suppl_data/Supplementary_Data4.zip!/Supplementary information 4 - plasma lipidomics.zip!/Plasma lipidomics/Control vs Patient/Statistical plots and data/PCA.pdf]

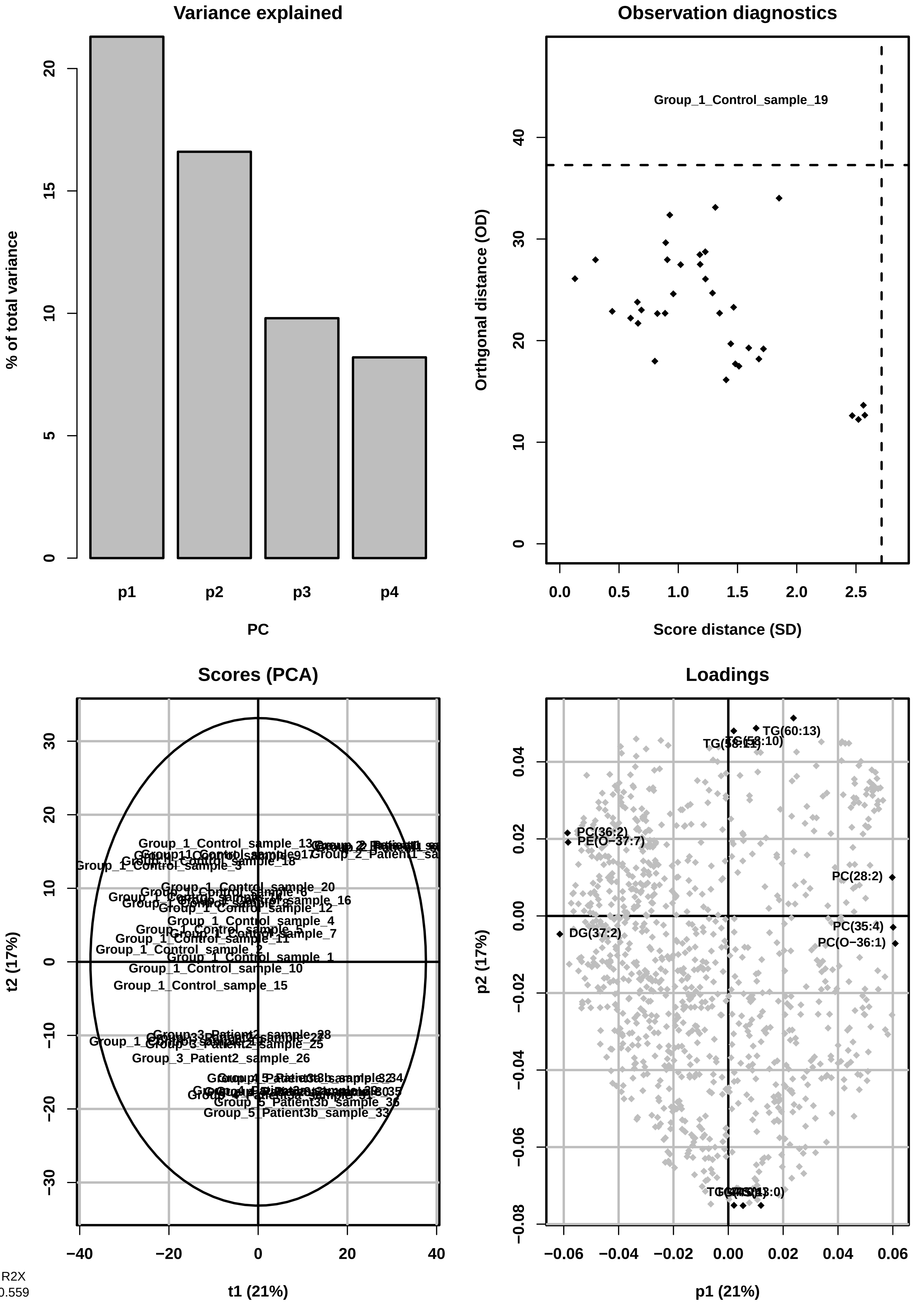
Variance explained
% of total variance
0
5
10
15
20
p1
p2
p3
p4
PC
Observation diagnostics
Group_1_Control_sample_19
Orthgonal distance (OD)
0
10
20
30
40
0.0
0.5
1.0
1.5
2.0
2.5
Score distance (SD)
Scores (PCA)
Group_1_Control_sample_13
Group_1_Control_sample_18
Group_1_Control_sample_3
Group_1_Control_sample_20
Group_1_Control_sample_16
Group_1_Control_sample_12
Group_1_Control_sample_4
Group_1_Control_sample_5
Group_1_Control_sample_7
Group_1_Control_sample_11
Group_1_Control_sample_2
Group_1_Control_sample_1
Group_1_Control_sample_10
Group_1_Control_sample_15
Group_3_Patient2_sample_28
Group_3_Patient2_sample_25
Group_3_Patient2_sample_26
Group_5_Patient3b_sample_36
Group_5_Patient3b_sample_33
t2 (17%)
t1 (21%)
30
20
10
0
−10
−20
−30
−40
−20
0
20
40
Loadings
TG(60:13)
PC(36:2)
PE(O−37:7)
PC(28:2)
PC(35:4)
PC(O−36:1)
DG(37:2)
p2 (17%)
p1 (21%)
0.04
0.02
0.00
−0.02
−0.04
−0.06
−0.08
−0.06
−0.04
−0.02
0.00
0.02
0.04
0.06
R2X
0.559
